# Evaluation of an In Situ Hardening *β*-Tricalcium Phosphate Graft Material for Alveolar Ridge Preservation. A Histomorphometric Animal Study in Pigs

**DOI:** 10.3390/dj6030027

**Published:** 2018-07-02

**Authors:** Minas Leventis, George Agrogiannis, Peter Fairbairn, Orestis Vasiliadis, Danai Papavasileiou, Evangelia Theodoropoulou, Robert Horowitz, Demos Kalyvas

**Affiliations:** 1 Laboratory of Experimental Surgery and Surgical Research N. S. Christeas, Medical School, University of Athens, 75 M. Assias Street, 115 27 Athens, Greece; orestis@vasiliadis.net (O.V.); eva_theod@yahoo.gr (E.T.); 2 Department of Pathology, Medical School, University of Athens, 75 M. Assias Street, 115 27 Athens, Greece; agrojohn@med.uoa.gr; 3 Department of Periodontology and Implant Dentistry, School of Dentistry, University of Detroit Mercy, 2700 Martin Luther King Jr Boulevard, Detroit, MI 48208, USA; peterdent66@aol.com; 4 Department of Oral and Maxillofacial Surgery, Dental School, University of Athens, 2 Thivon Street, 115 27 Athens, Greece; d.pap.mes@gmail.com (D.P.); demkal@dent.uoa.gr (D.K.); 5 Departments of Periodontics, Implant Dentistry, and Oral Surgery, New York University College of Dentistry, 345 E 24th Street, New York, NY 10010, USA; rah7@nyu.edu

**Keywords:** Alveolar ridge preservation, *β*-tricalcium phosphate, bone regeneration, bone substitutes, animal study

## Abstract

The purpose of this study was to investigate the effectiveness of a resorbable alloplastic in situ hardening bone grafting material for alveolar ridge preservation in a swine model. Seven Landrace pigs were used. In each animal, the maxillary left and right deciduous second molars were extracted, and extraction sites were either grafted with a resorbable alloplastic in situ hardening bone substitute, composed of beta-tricalcium phosphate (*β*-TCP) granules coated with poly(lactic-co-glycolic) acid (PLGA), or left unfilled to heal spontaneously. Animals were euthanized after 12 weeks, and the bone tissue was analyzed histologically and histomorphometrically. Linear changes of ridge width were also clinically measured and analyzed. Pronounced bone regeneration was found in both experimental and control sites, with no statistically significant differences. At the experimental sites, most of the alloplastic grafting material was resorbed and remnants of the graft particles were severely decreased in size. Moreover, experimental sites showed, in a statistically nonsignificant way, less mean horizontal dimensional reduction of the alveolar ridge (7.69%) compared to the control sites (8.86%). In conclusion, the *β*-TCP/PLGA biomaterial performed well as a biocompatible resorbable in situ hardening bone substitute when placed in intact extraction sockets in this animal model.

## 1. Introduction

Clinical trials and experimental preclinical studies have shown that the grafting of extraction sockets constitutes a predictable and reliable way to preserve the dimensions and architecture of the alveolar ridge [1,2,3,4,5,6]. Such measures involve the use of different kinds of bone grafts, barrier membranes and growth-factor preparations, and many different surgical techniques and protocols have been proposed [7,8,9,10]. Currently, it is still unclear which material or surgical method is the most effective in limiting post-extraction resorption while assisting in regenerating adequate vital bone. According to Yip et al. [11], the ideal bone grafting material should have specific attributes. It should be osteoconductive, osteoinductive and biocompatible. It is important to be gradually replaced by newly formed bone, exhibiting controlled breakdown and resorption, and it should be able to maintain the ridge contour in the augmented site. Moreover, it should have satisfactory mechanical properties and no risk of disease transmission.

In contemporary oral and implant surgery, bone substitutes are widely researched and utilized as an alternative to autogenous bone, in an attempt to avoid complicated procedures and reduce treatment time [12]. Alloplasts represent a group of synthetic and highly biocompatible bone grafting materials [13]. The use of alloplastic biomaterials does not pose a risk of transmitting infections or diseases, and their availability is unlimited [14,15,16]. Calcium phosphate ceramics are bioactive osteoconductive materials, and there is strong experimental evidence that they also have osteoinductive properties, while promoting neovascularization [17,18,19,20,21]. Among ceramics, beta-tricalcium phosphate (*β*-TCP) is widely used in orthopedics and in dentistry, mostly in the form of particulate grafts or cements. In order to improve its biological performance and mechanical properties, *β*-TCP can be combined with other synthetic materials [15,22,23,24,25,26,27,28,29]. Coating the *β*-TCP granules with poly(lactic-*co*-glycolic) acid (PLGA) produces an in situ hardening, stable, porous biomaterial that serves as a bioactive scaffold for bone reconstruction, while reducing the need for additional membranes to retain and stabilize the graft material in the bone defect [24,25,27].

Moreover, the increased stability throughout the grafted site, and the reduced micromotions of the graft particles might lead to enhanced bone regeneration. Such micromovements between bone and any implanted material may trigger differentiation of mesenchymal cells to fibroblasts instead of osteoblasts, and inhibit bone formation, resulting in the development of fibrous tissue [30,31,32].

The aim of this experimental study was to test the hypothesis that filling intact extraction sockets with a resorbable alloplastic in situ hardening biomaterial, consisting of *β*-TCP and PLGA, will limit resorption of the alveolar ridge, and assist in regeneration of new bone, compared to sites subjected to spontaneous healing, in a swine model.

## 2. Materials and Methods

### 2.1. Surgical Procedures

Seven 4-month-old female Landrace pigs, each weighing 18 kg (±2 kg), were used in this study with the approval of the Institutional Animal Care and Use Committee of the Veterinary Department, Greek Ministry of Rural Development and Veterinary, Attica Prefecture, Greece (8042/10-12-1014). The animals were allowed 7 days from their arrival to the N.S. Christeas animal research facility of the University of Athens Medical School, Athens, Greece to acclimatize to their new environment. Before and after operation were fed a balanced soft diet and water *ad libitum*, and caged individually in a standard manner.

**Figure 1:**
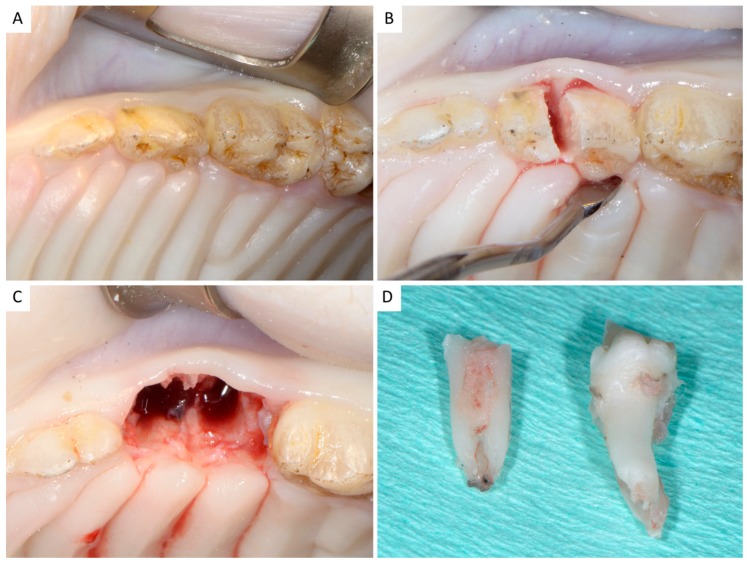
(**A**) Initial clinical view; (**B**) The deciduous maxillary second molar was sectioned, and each root was separately mobilized and extracted; (**C**) Clinical view of the site immediately after extraction; (**D**) The extracted tooth.

Animals were sedated with an intramuscular injection of 2 mg/kg body weight xylazine (Rompun, Bayer Hellas AG, Athens, Greece) plus 25 mg/kg body weight ketamine (Imalgene, Merial, Lyon, France). In each animal, the left and right second deciduous molars of the maxilla were “atraumatically” extracted without raising a flap (Figure 1, Video S1).

Intraoral local infiltrations with articaine and adrenaline 1:20,000 were performed buccally and palatally for hemostasis. Teeth were sectioned with a Lindemann bur under copious irrigation with sterile saline, each root was independently mobilized with periotomes, and removed with forceps, in order not to damage the surrounding soft and hard tissues, especially in the buccal aspect. Subsequently, the developing bud of the underlying second premolar, located at the apical part of the socket, was removed using a sharp Lucas bone curette. All sites were thoroughly debrided from soft tissues, and rinsed with sterile saline. The buccal bone plate in all cases had to be intact (4-wall extraction sockets).

**Figure 2:**
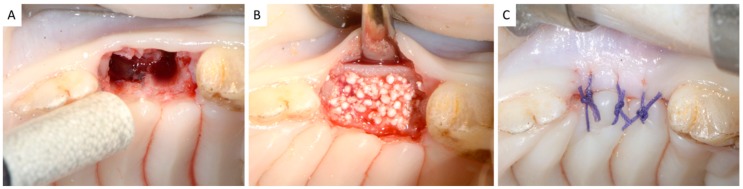
(**A**,**B**) The experimental sites were grafted with the resorbable alloplastic in situ hardening bone substitute; (**C**) All sites were closed without tension.

The extraction sites were either grafted (experimental sites, *n* = 10) or left unfilled (control sites, *n* = 4). A randomization technique using cards was followed. Paper cards were consecutively marked as “experiment” (*n* = 10) or “control” (*n* = 4). All cards were enclosed in identical sealed envelopes. Upon completion of each extraction, a clinical assistant not involved in the study was asked to open an envelope. A resorbable alloplastic in situ hardening bone grafting material (GUIDOR *easy-graft* CLASSIC, Sunstar Suisse SA, Etoy, Switzerland) was utilized to fill the experimental sites. This bone substitute is composed of *β*-TCP granules coated with a thin (10 μm) layer of PLGA. The biomaterial is preloaded in a sterile plastic syringe (Figure 2A), and a liquid Biolinker^TM^ (*N*-methyl-2-pyrrolidone solution) is mixed in the syringe with the graft granules prior to injecting the material into the socket. The Biolinker^TM^ turns the coated granules into a sticky mass, and allows moldability of the biomaterial, which begins to progressively harden in situ after application in the socket and upon contact with blood [28]. The graft granules were condensed in order to occupy the whole volume of the socket up to the level of the surrounding host bone (Figure 2B, Video S2), and the sites were closed in a tension-free manner with interrupted resorbable 3-0 sutures (Vicryl, Ethicon, Johnson & Johnson, Somerville, NJ, USA) (Figure 2C).

Each animal received intramuscularly antibiotics (enrofloxacin, Baytril 5%, Bayer Hellas AG, Athens, Greece) and analgesics (carpofen, Rimadyl, Pfizer Hellas SA, Athens, Greece) for 3 days postoperatively.

All animals were euthanized 12 weeks postoperatively with an intravenous injection of 100 mg/kg sodium thiopental (Pentothal, Abbott Hellas, Athens, Greece), and the parts of the jaws including the healed extraction sockets were surgically harvested.

At baseline and at 12 weeks, the width of the ridge was directly measured using a caliper 3 mm below the central part of the crest.

### 2.2. Histological and Histomorphometric Evaluation

The block specimens were fixed in 10% formalin for 2 days, and subsequently decalcified in bone decalcification solution (Diapath S.p.a., Martinengo, Italy) for 14 days. After routine processing, slices were obtained from the central part of the specimens using a saw (Exakt saw 312, Exakt Apparatebau GmbH, Norderstedt, Germany), embedded in paraffin, sectioned longitudinally into multiple 3 μm-thick sections, and stained with hematoxylin and eosin (H&E). For qualitative and morphologic analysis of the remodeling process, the stained preparations were examined under a light microscope (Nikon Eclipse 80, Nikon, Tokyo, Japan) at a minimum 20× magnification, and the entire section was evaluated. For histomorphometric analysis, images of each section were acquired with a digital camera microscope unit (Nikon DS-2MW, Nikon, Tokyo, Japan), and used to trace the areas identified as old maxillary bone, newly formed bone, residual graft, and connective tissue. A combination of Adobe PhotoShop (Adobe Systems Inc., San Jose, CA, USA) and image analysis software (Image-Pro Plus v. 5.1, Media Cybernetics, Rockville, MD, USA) was used to create individual layers of newly formed bone, biomaterial particles, and connective tissue. For each site, the following parameters were assessed: % new bone, % residual graft, % connective tissue, presence of inflammation, and complications. A single observer blinded to the clinical data carried out all analyses and measurements.

### 2.3. Statistical Analysis 

Statistical analysis was performed using SPSS software (v. 17, SPSS Inc., Chicago, IL, USA). Data were expressed as mean ± standard deviation (SD). The Kolmogorov–Smirnov test was used for normality analysis of the parameters. A comparison of variables between the two groups was performed using the independent samples *t*-test or Mann–Whitney test in case of violation of normality. Two-factor mixed factorial ANOVA was used to examine the interaction between the socket treatment factor and the time factor. Paired samples *t*-test was used for comparison of different time measurements of ridge width for each group. Comparison of percentage change of ridge width from initial evaluation to 12 weeks postoperative between the two groups was analyzed using the independent samples *t*-test or Mann–Whitney test in case of violation of normality. Using the analysis of covariance model (ANCOVA), the absolute change from initial assessment to 12 weeks postoperative of ridge width variable between the two groups was compared. All tests were two-sided, and statistical significance was set at *p* < 0.05.

## 3. Results

### 3.1. Overall

**Figure 3:**
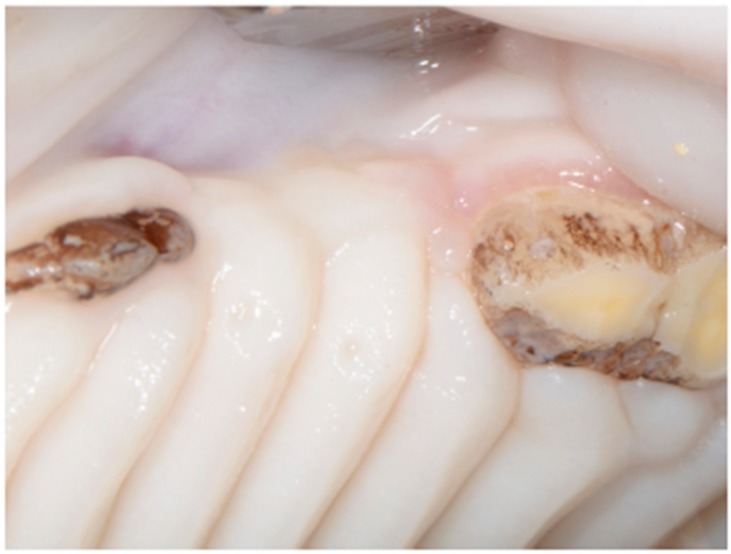
Normal healing after 12 weeks.

The postoperative course of all animals was uneventful. At 12 weeks, all sites were completely healed, covered by keratinized soft tissues (Figure 3). There were no clinical signs of local inflammation or infection.

### 3.2. Alveolar Ridge Dimensional Changes

After 12 weeks, all sites underwent several degrees of atrophy. The ridge width changed during the observation period in the same way for both groups (Table 1). Experimental extraction sites grafted with the alloplastic bone substitute showed less mean horizontal dimensional reduction of the alveolar ridge compared to sites subjected to spontaneous healing. However, this difference was not statistically significant (Table 2). 

### 3.3. Histology

**Figure 4:**
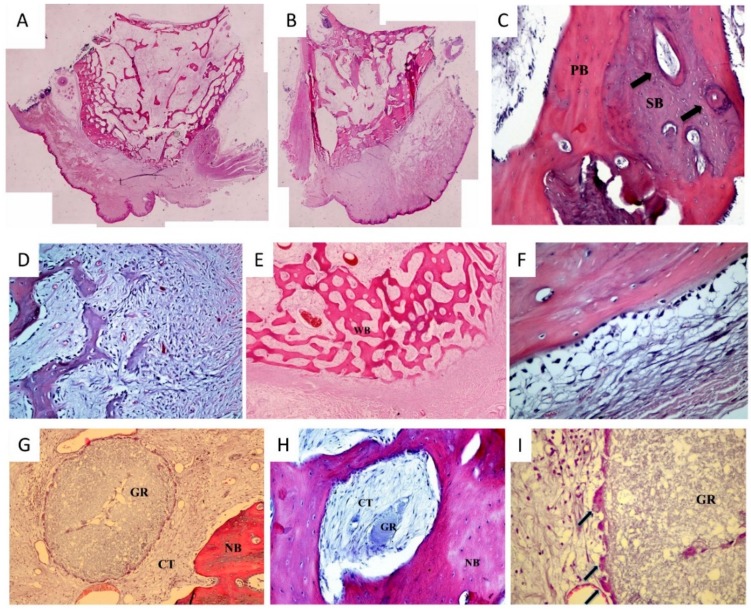
Representative histological pictures (H&E staining). (**A**) Site grafted with the resorbable alloplastic biomaterial at 12 weeks. The site is filled with regenerated bone, bone marrow and connective tissue. At the periphery of the socket, native cortical bone can be identified in continuity with the newly formed cancellous bone in the center. At this time point, most of the *easy-graft* CLASSIC particles have been resorbed; (**B**) Nongrafted extraction site at 12 weeks. Regenerated bone showing active remodeling; (**C**) At the center, mature secondary bone (SB) with harversian systems (arrows) can be observed, surrounded by less mature primary lamellar bone (PB). These types of bone are clearly separated by cement or reversal lines; (**D**) Newly formed bone trabeculae with osteoblast lining at the periphery, and connective tissue with numerous mesenchymal cells; (**E**) Newly formed woven bone (WB) sealing the entrance of the extraction socket; (**F**) Periosteum-like connective tissue covering the periphery of the socket with linear arrangement of osteoblasts; (**G**) Residual granule of *easy-graft* CLASSIC (GR) surrounded by connective tissue (CT) and newly formed bone (NB); (**H**) Remnant of *easy-graft* CLASSIC (GR) surrounded by a few multinucleated cells and connective tissue (CT) with blood vessels, fibroblasts, and collagen fibers. At the periphery, newly formed bone (NB) is present; (**I**) Multinuclear cells (arrows) lining the periphery of a granule of *easy-graft* CLASSIC (GR).

At 12 weeks, both experimental and control sites were filled with regenerated bone, bone marrow and connective tissue. At the periphery of all sockets, native cortical bone could be identified in continuity with the newly formed cancellous bone in the center. The entrances of the sockets were sealed with woven bone of immature state, which was covered by newly formed periosteum-like connective tissue with a linear arrangement of osteoblasts. Histologically, no soft tissue ingrowth was observed. All sockets were covered by regular stratified squamous keratinized epithelium. At the experimental sites, most of the alloplastic grafting material particles were resorbed, and the remnants were severely decreased in size (Figure 4).

### 3.4. Histomorphometry

The results of the histomorphometric analysis are shown in Table 3. At 12 weeks, more new bone formation was observed in sockets grafted with *easy-graft* CLASSIC compared to the sites that healed spontaneously. However, a statistically significant difference regarding osteogenesis was not demonstrated between the two groups. Experimental grafted sites exhibited small amounts of residual biomaterial at this time point.

## 4. Discussion

In this animal study, we investigated the effects of filling extraction sites in a swine model with a resorbable alloplastic in situ hardening bone grafting material.

After a 12-week healing period, all experimental sites healed uneventfully, with no clinical signs of local complications or infection. Histologically, the absence of acute inflammatory infiltrates and foreign body reactions confirms the good biocompatibility of the alloplastic biomaterial used.

The histologic and histomorphometric results showed pronounced new bone formation in both groups. Experimental sites, where ridge preservation measures were applied, showed more newly formed bone volume compared to the empty unassisted sites where natural healing occurred. However, our findings were not statistically significant. According to the current relevant literature, conflicting evidence exists on the benefit of alveolar ridge preservation techniques at the histologic level. Such techniques with the use of grafting materials do not appear to promote *de novo* hard tissue formation routinely. In addition, some graft materials may interfere with healing [1,3]. Grafting has been reported to impede the healing process in the fresh extraction socket model [31]. Biomaterials in a well-maintained space like the intact socket may arrest or delay bone formation, as it is reported that bone substitutes may modify the normal healing process and inhibit bone formation in grafted sockets compared to nongrafted sites [33,34,35].

In accordance with the present study’s observations, Leventis et al. [24], in a case series clinical study analyzing bone biopsies of human extraction sockets grafted with *easy-graft* CLASSIC, reported that after 4 months of healing, newly formed bone occupied 24.4% of the regenerated sites. These findings are similar to the results of the present study, where 20.33% of newly formed bone was observed at the animal extraction sockets at 12 weeks.

When regenerating alveolar bone, with the use of bone substitutes, the grafting material must have an appropriate resorption time in relation to new bone formation. It is also important to be replaced by host bone [11,36]. The long-term presence of residual nonresorbable or slowly resorbable particles of the graft might interfere with the bone healing mechanism and the remodeling of the new tissue, having a negative effect on the overall quality and architecture of the reconstructed bone. This is of great importance if the placement of a dental implant is planned, as bone-to-implant contact, initial implant stability and long-term function might be impaired in the presence of a high volume of nonresorbable graft particles in the regenerated bone tissue [36]. In the present study, the material used exhibited pronounced resorption after 12 weeks, being almost completely resorbed (0.26% of the tissue volume). Multinucleated giant cells were observed that seemed to be actively phagocytizing dissolved or partially dissolved graft granules. These findings confirm earlier animal studies showing fast resorption of *β*-TCP with time, and imply that the breakdown of *β*-TCP seems to be a combination of dissolution and direct cell-mediated resorption [34,37,38]. However, human extraction sockets grafted with *easy-graft* CLASSIC showed 12.9% residual biomaterial after 12 weeks [24].

The fast resorption of *easy-graft* CLASSIC shown in the present study can be explained by the fact that the material was placed into intact four-wall sockets, meaning that the high vascularity from the surrounding thick bone plates might have resulted in pronounced resorption by multinucleated phagocytes derived from the host blood. The findings could also be explained by the higher metabolic activity of the animal model used in the present study. Finally, these findings of pronounced graft resorption could be attributed to washout of some material from the grafted socket during healing due to friction induced by mastication of the pig. However, the biomechanical in situ hardening characteristics of *easy-graft* CLASSIC provide adequate stability to the material as the granules adhere to each other. In this respect, they form a hard scaffold that interlocks into the defect, and therefore it can be left uncovered to heal by secondary intention. Data from human clinical studies and case reports show that the self-hardening characteristics of such alloplastic grafting materials facilitate the secondary healing of grafted sockets, without evidence of washout of the uncovered biomaterial in the oral environment [24,26,39]. Moreover, in the present study, the grafted sites were sutured tension-free, so no material was exposed postoperatively. However, in contrast to human studies, in this experimental animal model, it was not possible to monitor and observe clinically any wound dehiscence and/or washout of grafting material during the healing period. Clinical observations were possible only on the day of surgery and the day of euthanasia. In order to clinically evaluate the extraction sites at any other time point in between, strong medical sedation of the animals would be necessary, which could threaten their health.

Tooth extraction not only results in resorption of the alveolar bone, but also triggers a structural change in the overlying soft tissue [40]. Human studies reporting data on horizontal hard and soft tissue changes have shown a reduction of 0.1–3.8 mm, with a weighted mean reduction of 1.3 mm, after extraction without applying any grafting procedures [40]. Recent systematic reviews have demonstrated that alveolar ridge preservation techniques may contribute to less ridge width reduction than what occurs following natural socket healing, although such techniques do not totally eliminate post-extraction resorption [1,2,3,4].

In this study, we measured in each group the combined hard and soft tissue change in the horizontal dimension, following extraction, in order to evaluate the alterations of overall ridge width. Comparing the absolute change of the horizontal dimension from baseline to 12 weeks postoperatively between the two groups, the present results showed that the experimental grafted sites underwent less resorption than the control nongrafted sites. However, the differences between the two groups were not statistically significant. It is important that in the present study, similar to other studies [40], the overall dimensions of the alveolar ridge were analyzed corresponding to alterations of the soft and hard tissues. It is stated that the soft tissue may increase in dimension to partially compensate for the resorption of the hard tissue. For this reason, such assessments may not be appropriate to evaluate the effectiveness of ridge preservation procedures regarding the alveolar bone [4,41]. In the present study, only linear measurements of the width of the ridge were made. Volumetric measurements of the buccal and occlusal volumes would provide more accurate data regarding the loss of volume and changes of the ridge profile in both groups.

The statistically nonsignificant results in the present study may be attributed to several reasons. A larger sample could have resulted in statistically significant results; however, for ethical reasons and to comply with the guidelines of the ethical committee, the smallest number of animals was used in the study. Another explanation could be the minimally invasive surgical technique utilized and the use of four-wall sockets with an intact thick buccal bone. An important factor determining the quality of the socket after extraction is the presence of the buccal wall, meaning that these sites have high regenerative potential and show less resorption even following natural spontaneous healing without the aid of Guided Bone Regeneration measures [42]. In contrast, sockets with thin (1 mm or less) or defective fenestrated buccal walls are prone to more atrophy, up to 56% of horizontal resorption [43,44]. A thin cortical buccal plate has poor vascularity, and contains few marrow spaces. So, after extraction and especially when a buccal full-thickness flap is raised, which would compromise the blood supply from the periosteum more, it will resorb quickly, resulting in pronounced atrophy of the site. As shown in a different experimental study, it can be postulated that the effect of grafting the extraction sites with *easy-graft* would be more pronounced if sockets with thin, defective, or missing buccal plates were treated [45]. It is reasonable that in such defective sites, where more pronounced atrophy is expected after spontaneous natural healing, the above grafting measures may have a greater impact on preserving the width of the ridge, and the possible differences between the experimental and control groups might be more significant and conclusive.

## 5. Conclusions

Within the limits of the present experimental study, it can be concluded that the resorbable alloplastic bone substitute showed excellent biocompatibility and can support new bone formation when placed in intact extraction sockets for alveolar ridge preservation in this animal model. The use of *easy-graft* CLASSIC did not impede the healing of fresh four-wall extraction sites, as reported for other bone graft substitutes, while the in situ hardening characteristics of the material can improve the handling and stability of the sites, allowing for minimally invasive procedures.

## Figures and Tables

**Table 1:** Comparison of absolute values of alveolar ridge width between groups at each time point.

	*N*	Mean (mm)	SD	*p*-Value
Initial				
Graft	10	7.93	0.44	0.319
Control	4	7.63	0.63	
12 weeks postop				
Graft	10	7.32	0.59	0.233
Control	4	6.93	0.29	

**Table 2:** Evaluation of percentage change of ridge width from initial to 12 weeks postoperative for experimental and control sites.

	*N*	Mean (%)	SD	*p*-Value
Initial to 12 weeks postop				
Graft	10	−7.69	5.46	0.727
Control	4	−8.86	5.92	

**Table 3:** Percentages of new bone, connective tissue and residual graft occupying the sockets.

Parameter	Group	*N*	Mean	SD	*p*-Value
New bone %	Graft	10	20.33	8.10	0.268
	Control	4	15.40	3.01	
Connective tissue %	Graft	10	76.24	10.01	0.198
	Control	4	83.26	1.63	
Residual graft %	Graft	10	0.26	0.38	-
	Control	4	-	-	

## Supplementary Materials

The following are available online at http://www.mdpi.com/2304-6767/6/3/27/s1: Video S1: Minimally invasive extraction of the deciduous maxillary second molar. Video S2: Grafting of the extraction site (experimental group) with the in situ hardening alloplastic biomaterial.
